# 2023 Updated MASCC/ESMO Consensus Recommendations: prevention of radiotherapy- and chemoradiotherapy-induced nausea and vomiting

**DOI:** 10.1007/s00520-023-08226-z

**Published:** 2023-12-15

**Authors:** Christina H. Ruhlmann, Karin Jordan, Franziska Jahn, Ernesto Maranzano, Alex Molassiotis, Kristopher Dennis

**Affiliations:** 1 Department of Oncology, Odense University Hospital, Odense, Denmark; 2 Department of Clinical Research, University of Southern Denmark, Odense, Denmark; 3 Department for Hematology, Oncology and Palliative Medicine, Ernst von Bergmann Hospital Potsdam, Potsdam, Germany; 4 Department of Medicine V, Hematology, Oncology and Rheumatology, University of Heidelberg, Heidelberg, Germany; 5 Clinic for Internal Medicine IV, Oncology–Hematology–Hemostaseology, University Hospital Halle (Saale), Halle, Germany; 6 University of Perugia, Perugia, Italy; 7 College of Arts, Humanities and Education, University of Derby, Derby, UK; 8 The Ottawa Hospital and the University of Ottawa, Ottawa, Canada

**Keywords:** Antiemetic, Concomitant chemotherapy, Guideline, Nausea, Radiotherapy, Vomiting

## Abstract

**Purpose:**

Radiotherapy and chemoradiotherapy-induced nausea and vomiting (RINV and C-RINV) are common and distressing, and there is a need for guidance for clinicians to provide up to date optimal antiemetic prophylaxis and treatment. Through a comprehensive review of the literature concerning RINV and C-RINV, this manuscript aims to update the evidence for antiemetic prophylaxis and rescue therapy and provide a new edition of recommendations for the MASCC/ESMO antiemetic guidelines for RINV and C-RINV.

**Methods:**

A systematic review of the literature including data published from May 1, 2015, to January 31, 2023, was performed. All authors assessed the literature.

**Results:**

The searches yielded 343 references; 37 met criteria for full article review, and 20 were ultimately retained. Only one randomized study in chemoradiation had the impact to provide new recommendations for the antiemetic guideline. Based on expert consensus, it was decided to change the recommendation for the “low emetic risk” category from “prophylaxis or rescue” to “rescue” only, while the drugs of choice remain unchanged.

**Conclusion:**

As for the previous guideline, the serotonin receptor antagonists are still the cornerstone in antiemetic prophylaxis of nausea and vomiting induced by high and moderate emetic risk radiotherapy. The guideline update provides new recommendation for the management of C-RINV for radiotherapy and concomitant weekly cisplatin. To avoid overtreatment, antiemetic prophylaxis is no longer recommended for the “low emetic risk” category.

## Introduction

Radiotherapy is an important treatment modality offered to approximately 50% of patients with cancer in either the curative or palliative setting [1]. Radiotherapy-induced nausea and vomiting (RINV) are common and often undertreated symptoms among patients receiving radiotherapy, and the risk varies with the different sites of irradiation and the delivered radiation dose per fraction [2]. Hence, it is important that clinicians know how to prevent or ameliorate nausea and vomiting in different radiotherapy settings, ensuring that patients complete the treatment successfully without critical dose delays and maintaining optimal quality of life.

This is an update of the Multinational Association of Supportive Care in Cancer (MASCC) and European Society for Medical Oncology (ESMO) antiemetic guideline for radiotherapy update 2015 [3], part of the 2015 MASCC and ESMO guideline update for the prevention of chemotherapy- and radiotherapy-induced nausea and vomiting and of nausea and vomiting in cancer patients [4]. The purpose of the update is to review the literature of clinical trials in radiotherapy or concomitant chemoradiotherapy from 2015 to present and based on the literature to provide an update of the evidence-based guideline for the use of antiemetic prophylaxis and treatment in radiotherapy or concomitant chemoradiotherapy.

## Literature review and methods

A medical librarian searched Ovid Medline, the Cochrane Central Register of Controlled Trials, Embase Classic, and Embase for references pertaining to RINV and C-RINV without restrictions on the type of study. An initial search was conducted on 8 July 2022 for references published from 1 May 2015 to 8 July 2022, and an updated identical search was performed on 21 July 2023 for references published from 9 July 2022 to 31 Jan 2023. The total review period thus extended from 1 May 2015 to 31 Jan 2023. Two members of the committee (KD and CR) screened all titles and abstracts of the references from the search to identify those requiring full article review. References were excluded if the studies were not focused on nausea and vomiting experienced by patients receiving radiotherapy or concomitant chemoradiotherapy, if they covered pediatric patients or if they were written in a language other than English.

All authors assessed the included literature for full text review. Three web meetings and several email correspondences with discussions and conclusions preceded the final proposal for the RINV guideline update, which was presented and finally approved by the MASCC/ESMO Antiemetic Guidelines Consensus Committee.

## Results

**Fig. 1:**
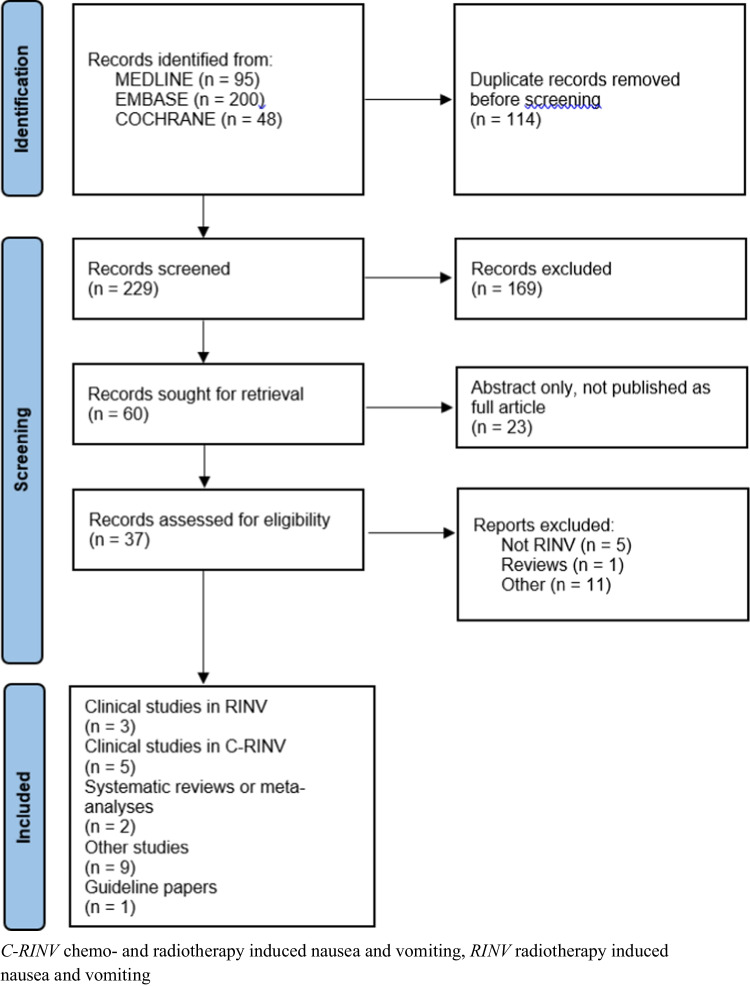
PRISMA Flow Diagram of combined 8 July 2022 and 21 July 2023 literature search results

The combined searches yielded 343 references (321 from the first search and 22 from the second); 114 duplicates were removed (113 + 1), leaving 229 total references for screening (208 + 21). Of the 229, 169 were excluded during screening leaving 60 references for which the full articles were reviewed. Of the 60, 23 were published as abstracts only, leaving 37 articles retained for full article review. Of the 37, 16 records were excluded after full article review for various reasons (e.g., not RINV relevant, lower methodology, and reviews), leaving 20 publications being finally included for potential incorporation into update recommendations (Fig. 1). Three publications were classified as RINV clinical trials or prospective studies [5–7]; one study was a meta-analysis [8]; 12 publications addressed concomitant chemoradiotherapy including two phase III antiemetic clinical trial [9–20]; and five studies concerned risk factors, practice patterns, methodology in RINV clinical trials, and other [4, 21–23]. The studies are reviewed and discussed below.

### Risk classification

#### Risk factors

Risk factors for RINV are less investigated compared to those for chemotherapy-induced nausea and vomiting (CINV). Two observational studies by the Italian Group for Antiemetic Research in Radiotherapy (IGAAR) identified that irradiated site (upper abdomen), field size > 400 cm^2^, and concomitant chemotherapy are independent risk factors for development of RINV [2, 24].

Since the 2015 update, none of the published data have provided results for patient or treatment related risk factors to modify the risk classification guideline. In a small retrospective study (*n* = 62) by Uno et al., risk factors associated with nausea and vomiting in patients with cervical cancer receiving radiotherapy with or without concomitant weekly cisplatin (40 mg/m^2^) were explored [23]. Patients treated with cisplatin received granisetron and dexamethasone as antiemetic prophylaxis. In summary, patients aged > 65 years had clinically significantly less nausea and vomiting compared to the younger patients, and the risk difference was regardless of concomitant cisplatin or radiotherapy alone. Only 27% of the younger patients in the concomitant cisplatin group achieved complete response (no vomiting and no use of rescue medication). The study suggests that younger patients treated for cervical cancer with radiotherapy alone should be considered for antiemetic prophylaxis, and as demonstrated in the GAND-emesis study [10], patients with cervical cancer (regardless of age) treated with radiotherapy and concomitant weekly cisplatin should receive antiemetic prophylaxis with a neurokinin (NK)_1_-receptor antagonist (RA), a 5-hydroxytryptamine(5-HT)_3_-RA, and dexamethasone.

#### Levels of emetic risk with radiotherapy

The emetic risk of radiotherapy is divided into four risk levels; high, moderate, low, and minimal (Table 1). The risk levels are categorized according to the site of irradiation with different emetic risk potentials. The emetic risk of the four levels is based on observations from clinical trials, cohort studies, and expert opinions. The emetic risk levels of the various sites of radiation remain the same as for the previous guideline update. The incidence of emesis (proportion of patients with emesis if no antiemetics are provided) of the four risk levels is poorly described, however well described for total body irradiation and half body irradiation [25, 26]. In the 2009 edition of the guideline, and mainly based on expert opinions, percentages for emetic risk were displayed for the four levels. However, acknowledging the high uncertainty of the percentages and the fact that the figures do not influence the guideline recommendations, it was decided for the 2015 update to omit the percentages. Conversely, the American Society of Clinical Oncology (ASCO) antiemetic guideline continues to display the percentages of the four risk categories [27].

**Table 1:** Radiotherapy emetic risk levels and MASCC/ESMO antiemetic guideline update 2023

Emetic risk level	Area of treatment	Antiemetic guideline	Level of evidence/grade of recommendation
High	Total body irradiation	Prophylaxis with a 5-HT_3_-RA + DEX	II/B (for the addition of DEX: III/C)
Moderate	Upper abdomen, craniospinal	Prophylaxis with a 5-HT_3_-RA + optional DEX	II/A (for the addition of DEX: II/C)
Low	Brain	Rescue with DEX	IV/B
	Head & neck, thorax, pelvis	Rescue with DEX, a dopamine RA, or a 5-HT_3_-RA	IV/B
Minimal	Extremities, breast	Rescue with DEX, a dopamine RA, or a 5-HT_3_-RA	IV/B
Concomitant RT and weekly cisplatin 40 mg/m^2^	Acute NV: prophylaxis day 1 before administration of cisplatin with a 5-HT_3_-RA, DEX, and an NK_1_-RA Delayed NV: DEX days 2-4		II/B
Concomitant CRT	In concomitant CRT, the antiemetic prophylaxis is according to the CT-related antiemetic guidelines of the corresponding risk category; unless, the risk of emesis is higher with RT than CT		IV/B

Dexamethasone on days 2–4 was administered in the clinical trial including cervical cancer patients (younger women, pelvic radiation). No trial has compared dexamethasone day 1 vs days 2–4, and no trial has explored the optimal regimen in other treatment settings

*5-HT*_*3*_*-RA* 5-hydroxytryptamine_3_-receptor antagonist, *CT* chemotherapy, *CRT* chemoradiotherapy, *DEX* dexamethasone, *NV* nausea and vomiting, *RT* radiotherapy

### Antiemetic efficacy studies in radiotherapy

Three RINV clinical trials and one meta-analysis in patients receiving single fraction or fractionated radiotherapy are discussed.

Due to the low incidence of RINV for the “low emetic risk” level, no high-quality studies have investigated the use of prophylaxis in this setting. The guideline expert panel estimated that the majority of patients will be subjected to overtreatment if using prophylaxis. Therefore, it was decided to adjust the recommendation for the “low emetic risk” level to recommend rescue antiemetics only.

#### Efficacy of 5-HT_3_ receptor antagonists

As for previous updates of the MASCC/ESMO antiemetic guideline for radiotherapy, no specific 5-HT_3_-RA as antiemetic prophylaxis or rescue treatment is recommended over another.

A meta-analysis published in 2017 assessed 17 randomized controlled trials for efficacy of antiemetic regimens in radiotherapy [8]. Among patients receiving radiotherapy to the abdomen/pelvis, the study found that prophylaxis with a 5-HT_3_-RAs was significantly more efficacious than placebo and dopamine-RAs in both complete control of vomiting [OR 0.49; 95% confidence interval (CI), 0.33–0.72 and OR 0.17; 95% CI, 0.05–0.58 respectively] and complete control of nausea (OR 0.43; 95% CI, 0.26–0.70 and OR 0.46; 95% CI, 0.24–0.88 respectively). Prophylaxis with 5-HT_3_-RAs was also more efficacious than rescue therapy and dopamine RAs plus dexamethasone. The addition of dexamethasone to 5-HT_3_-RAs compared to 5-HT_3_-RAs alone provides a modest improvement in prophylaxis of RINV. Among patients receiving total body irradiation, 5-HT_3_-RAs were more effective than other agents (placebo, combination of metoclopramide, dexamethasone, and lorazepam). These findings are in accordance with the 2015 guideline update, which remains unchanged in the current update.

Palonosetron as RINV prophylaxis was explored in a pilot study including 75 patients receiving low or moderate emetic risk radiotherapy in a palliative setting (8 Gy single fraction (*n* = 44), 20 Gy in 5 fractions, or 30 Gy in 10 fractions) [5]. Patients received 0.5 mg of palonosetron orally, at least one hour prior to the first fraction of radiotherapy, and every other day until treatment completion. Complete control (no emetic episode, no use of rescue medication, and no more than mild nausea) was the primary efficacy parameter and results were compared with historical data. In the acute phase (day 1 of treatment to day 1 post-treatment), 93.3% and 74.7% reported complete control of vomiting and nausea, respectively. In the delayed phase (days 2–10 post-treatment), 93.2% and 74.0% reported complete control of vomiting and nausea, respectively. These figures were clinically significantly higher compared to a historical cohort using ondansetron. The results need to be confirmed in a larger scale randomized setting to assess the efficacy and tolerability of multiple doses of palonosetron, and the potentially modulating effect of dexamethasone which is often given for the purpose of pain flare prophylaxis among patients undergoing radiotherapy for bone metastases.

#### Efficacy of NK_1_-receptor antagonists

The NK_1_-RAs as antiemetic prophylaxis in radiotherapy remains largely unexplored.

Two small clinical studies including NK_1_-RAs for the prevention of RINV have been published. One study randomized patients scheduled to receive radiotherapy with at least 30 Gy in total to receive either ondansetron (*n* = 20) or ondansetron plus aprepitant (*n* = 20) [6]. However, 80% in the combination group received concomitant chemotherapy, whereas the figure was 60% for the ondansetron only group. There is no information on the antiemetics provided for CINV. The endpoint was symptoms of RINV, unspecified, and results showed significantly higher grade of RINV for the ondansetron group compared to the combination group.

The other study was a phase II single arm study including 52 evaluable patients receiving radiotherapy to the upper abdomen [7]. Patients receiving fractionated radiotherapy (at least 40 Gy in total) with or without radiosensitizing chemotherapy received oral ondansetron 8 mg BID and aprepitant 125/80/80 mg on Monday, Wednesday, and Friday throughout radiotherapy. Complete response (no vomiting, no use of rescue therapy) during the entire observation period of radiotherapy was achieved by 57.7% (30/52; 95% CI, 43.2–71.3%). Nausea was common with 61.5% reporting significant nausea at any time during the observational period. Compared to historical data, aprepitant and ondansetron as dosed in this trial were not superior to standard ondansetron monotherapy.

From a methodological point of view, it is difficult to draw conclusions from these studies regarding efficacy of addition of aprepitant for the prevention of RINV, and the research question about efficacy of NK_1_-RAs for the prevention of RINV remains unanswered.

#### Effects of integrative and complementary therapies on RINV

Integrative oncology (i.e., the use of mind and body practices, natural products, and/or lifestyle modifications, etc.) is extensively explored for the reduction of CINV, whereas for RINV only a few studies have attempted to but failed in demonstrating efficacy of e.g. acupuncture [28]. Thus, Enblom et al. have been looking into the use of integrative oncology techniques and conducted a survey in 200 patients treated with abdominal/pelvic irradiation [22]. Daily registrations of nausea and practice of complementary self-care strategies were collected. Two thirds of the patients experienced nausea, and 25% practiced self-care for nausea at least once, mostly by modifying eating or drinking habits, for a mean of 15.9 days. Interestingly, patients who practiced integrative self-care experienced less nausea.

### Antiemetic efficacy studies in chemoradiotherapy

The findings in an observational study in patients receiving radiotherapy and concomitant low-dose cisplatin, comparing two cohorts using either antiemetic prophylaxis with a 5-HT_3_-RAs and dexamethasone (control) or the same prophylaxis plus aprepitant, demonstrated a trend towards higher control rates for nausea and vomiting in patients receiving the NK_1_-RA [9].

The GAND-emesis study was a well-designed phase III trial comparing fosaprepitant 150 mg day 1 with placebo both combined with palonosetron and dexamethasone for the prevention of chemoradiotherapy induced nausea and vomiting (C-RINV) in cervical cancer patients (*n* = 246) treated with fractionated radiotherapy and concomitant weekly cisplatin 40 mg/m^2^ [10]. The primary endpoint was the “sustained no emesis” rate (SNE; complete free from emesis during five weeks of chemoradiotherapy). The study found a SNE rate of 49% for the placebo group compared with 66% for the fosaprepitant group (subhazard ratio 0.58 [95% CI, 0.39–0.87]; *p*=0.008). The study proved the superiority of addition of an NK_1_-RA to a 5-HT_3_–RA and dexamethasone in the setting of low-dose cisplatin concomitant to radiotherapy.

Olanzapine (10 mg daily days 1–5) compared to fosaprepitant (150 mg day 1), both in combination with palonosetron and dexamethasone, was explored in a placebo-controlled clinical trial in patients treated for locally advanced head and neck cancer or locally advanced esophageal cancer receiving radiotherapy and concomitant cisplatin > 70 mg/m^2^ and 5-fluorouracil, 750 mg/m^2^ a day for 4 days [11]. Efficacy was assessed only for the 120 hours following the first cycle of chemotherapy, and the primary endpoint was complete response overall (120 hours), for which there was no difference between groups (76% and 74% for the olanzapine and fosaprepitant groups, respectively). Due to the study design, the study reports on CINV rather than RINV.

A small single arm study in cervical cancer patients treated with fractionated radiotherapy and concomitant weekly cisplatin 40 mg/m^2^ analyzed 65 patients receiving weekly antiemetic prophylaxis with oral olanzapine 5 mg days 1 and 2, intravenous palonosetron 0.25 mg day 1, and intravenous dexamethasone 12 mg day 1 [12]. The complete response rate was 55%; no vomiting and no nausea were achieved by 63% and 46%, respectively. The time frame for the endpoint is unclear, and the use of NK_1_-RA as rescue is not shown. The use of olanzapine as prophylaxis without an NK_1_-RA for C-RINV in the described setting cannot be recommended.

Two small single arm studies evaluated antiemetic prophylaxis in patients with cervical cancer receiving fractionated radiotherapy and concomitant daily low-dose cisplatin 8 mg/m^2^. The first study (*n* = 27) evaluated the efficacy of weekly, day 1 administration of intravenous palonosetron 0.75 mg plus oral aprepitant (125 mg day 1, 80 mg days 2–3). Dexamethasone was only used as rescue [13]. The primary efficacy endpoint complete response (no emetic episodes and no rescue medication during the complete treatment period) was achieved by 48%. Rescue medication was needed for 52% of the patients. The second study (*n* = 26) evaluated the efficacy of weekly, day 1 administration of intravenous palonosetron 0.75 mg and oral dexamethasone (2 mg twice daily) from day 1 to the end of the treatment period [14]. Complete response, as defined for the previous study, was achieved by all 100% of the patients. In conclusion, these studies highlight the need for adherence to applicable existing guidelines to avoid potential under- or overtreatment, but also the need for further investigation of optimal antiemetic regimens for low-dose daily cisplatin 8 mg/m^2^ concomitant to radiotherapy.

Two small prospective studies evaluated the safety and efficacy of antiemetics in patients with malignant glioma receiving standard radiotherapy and concomitant temozolomide (TMZ). The first study (*n* = 38) evaluated a weekly dose of intravenous palonosetron 0.25 mg for up to 6 weeks [15]. C-RINV complete response rates (no vomiting and no use of rescue antiemetics) for 6 weeks ranged from 67 to 79%. The second study (*n* = 21) evaluated the addition of aprepitant to palonosetron and dexamethasone [16]. Complete response rate in the overall period was 76%, and comparing to a historical cohort using a 5-HT_3_ receptor antagonist and dexamethasone, the addition of aprepitant significantly improved the complete response rate. Results need to be confirmed in larger scale comparative trials.

Patients (*n* = 43) scheduled for fractionated radiotherapy and concomitant cisplatin 100 mg/m^2^ (33 mg/m^2^ days 1–3) every 3 weeks for two cycles were prospectively assessed for efficacy of an antiemetic prophylaxis regimen consisting of oral aprepitant 125 mg day 1, 80 mg days 2–5; intravenous ondansetron 8 mg day 1; and oral dexamethasone 12 mg day 1, 8 mg on days 2–5 [17]. The antiemetics were provided for each chemotherapy cycle, and 37 patients completed the two planned cycles. The complete response rate for the overall period was 86%. The study assessed CINV rather than RINV.

A prospective cohort study (*n* = 33) assessed the risk of C-RINV during neoadjuvant long-course radiation therapy (low emetic potential) and concurrent 5-fluorouracil-based chemotherapy (low emetic potential) for rectal adenocarcinoma [18]. No antiemetic prophylaxis was used. The co-primary outcome “vomiting during the entire course of radiotherapy” was observed in 18% of the patients, and one third of the patients used rescue antiemetics during the treatment. Nausea occurred in 64% of the patients during the treatment course, and the onset of nausea was at median 7 days as opposed to 20 days for time to first vomiting episode. The study, subject to a low sample size, underlines the rationale for providing rescue antiemetics for the specific treatment indication, as prophylaxis would result in substantial overtreatment.

## Guideline recommendations: update 2023

1. Recommendation 1; High emetic risk: Patients receiving radiotherapy at a high emetic risk level should receive prophylaxis with a 5-HT_3_-RA plus dexamethasone. Level of Evidence: II; Grade of Recommendation: B (for the addition of dexamethasone: III/C).
2. Recommendation 2; Moderate emetic risk: Patients receiving radiotherapy at a moderate emetic risk level should receive prophylaxis with a 5-HT_3_-RA and optional short course dexamethasone. Level of Evidence: II; Grade of Recommendation: A (for the addition of dexamethasone: II/C).
3. Recommendation 3; Low emetic risk: No routine primary prophylaxis is suggested. Patients receiving radiation therapy of the brain should receive rescue therapy with dexamethasone. Patients receiving radiation therapy to head & neck, thorax, or pelvic sites should receive rescue with dexamethasone, a dopamine-RA, or a 5-HT_3_-RA. Level of Evidence: IV; Grade of recommendation: B.
4. Recommendation 4; Minimal emetic risk: No routine primary prophylaxis is suggested. Patients receiving radiotherapy at a minimal emetic risk level should receive rescue with dexamethasone, a dopamine-RA, or a 5-HT_3_-RA. Level of Evidence: IV; Grade of Recommendation: B.
5. Recommendation 5; Radiotherapy/weekly cisplatin: Patients receiving radiotherapy and concomitant weekly cisplatin should receive prophylaxis before cisplatin administration with a three-drug regimen including a 5-HT_3_-RA, dexamethasone, and fosaprepitant/aprepitant for the prevention of acute nausea and vomiting. Level of Evidence: II; Grade of Recommendation: B.
6. Recommendation 6; Radiotherapy/weekly cisplatin: In patients receiving radiotherapy and concomitant weekly cisplatin treated with a 5-HT_3_-RA, dexamethasone, and fosaprepitant/aprepitant for the prevention of acute nausea and vomiting, dexamethasone on days 2 to 4 is suggested to prevent delayed nausea and vomiting. Level of Evidence: II; Grade of Recommendation: B.
7. Recommendation 7; Concomitant radio-chemotherapy: Patients receiving concomitant radio-chemotherapy should receive antiemetic prophylaxis according to the chemotherapy-related antiemetic guidelines of the corresponding risk category, unless the risk of nausea and vomiting is higher with radiotherapy than with chemotherapy. Level of Evidence: IV; Grade of Recommendation: B.

## Discussion

The systematic literature review provided the basis for an evidence-based update of the recommendations for RINV and C-RINV management. However, the evidence for management of, especially, low and minimal emetogenic radiotherapy remains very limited. For high and moderate emetic level, the evidence for the recommendations is higher (II, B and II, A), and the cornerstone in this setting is still a 5-HT_3_-RA ± dexamethasone. Contributing to the level of evidence, a meta-analysis analyzed 17 randomized studies in RINV [8]. However, the studies often apply different methodologies (e.g., different primary endpoints, time frames, and antiemetic schedules), and the study heterogeneity complicates the comparison and introduces risk of bias. This inconsistency in study design has been addressed in a systematic review of methodologies, endpoints, and outcome measures in 34 randomized studies of RINV [21]. Of special notice, only 29% of the randomized studies had a primary endpoint a priori. It is clear that there is a need for scientifically high-quality research in RINV, and the authors call for recommendations for ideal trial design and reporting.

There is a need for further improvement of the control of RINV in highly and moderately emetic risk settings. There is no preferred 5-HT_3_-RA for prophylaxis or rescue. A small study has explored the use of the 5-HT_3_-RA palonosetron and compared to historical data [5]. There seems to be improved control of RINV when palonosetron is used compared to ondansetron. However, there is a need for larger prospective trials to assess the efficacy, safety, and impact on quality of life of palonosetron in this setting. This is also the case for NK_1_-RAs which are not part of the guideline for the prevention of RINV. One small single arm study explored aprepitant as prophylaxis in the moderately emetic risk category and found that the response rates were comparable to historical cohorts not using an NK_1_-RA [7]. Further investigation of the NK_1_-RAs in selected treatment settings is warranted.

Nausea is one of the most distressing symptoms in patients receiving chemoradiotherapy including weekly cisplatin [20]. In this setting, progress has been made, and a new recommendation incorporated in the current guideline is a triple antiemetic regimen including an NK_1_-RA (fosaprepitant/aprepitant), a 5-HT_3_-RA, and dexamethasone [10]. Based on this regimen, patients will encounter less nausea (15% with no nausea during the 5 weeks of treatment compared to 8% in the placebo/no NK_1_-RA group), and further investigations to specify the group that might benefit from further anti-nausea agents (e.g., olanzapine) are needed. The guideline update recommends specifically the NK_1_-RA fosaprepitant/aprepitant for weekly administration. The NK_1_-RAs rolapitant and netupitant have considerable longer plasma half-lives (approximately 180 and 88 hours, respectively) compared to fosaprepitant/aprepitant (approximately 9–13 hours), and the safety during weekly administration is unclear. A prospective study investigating the safety of NEPA (netupitant and palonosetron) during weekly administration for 5 weeks in patients receiving fractionated radiotherapy and concomitant weekly cisplatin in ongoing (NCT03668639).

RINV and C-RINV continue to have an impact on patients quality of life. A cross-sectional multinational survey among physicians, nurses, and patients showed that the health care professionals overestimated the incidence of C-RINV but underestimated the impact that this had on patients’ daily lives [19]. Knowledge sharing and guideline dissemination are important in order to provide evidence-based antiemetic treatment to our patients worldwide.

## Conclusion

In summary, none of the published data on RINV since 2015 has influenced the current update of the RINV antiemetics recommendations. However, in concomitant chemoradiotherapy, a single study was identified to impact the guidelines update for C-RINV [10], providing specific recommendations for prophylaxis during weekly cisplatin 40 mg/m^2^ concomitant to fractionated radiotherapy. Moreover, the recommendation for the RINV “low emetic risk” category was changed from “prophylaxis or rescue” to “rescue” only, while the drugs of choice remain unchanged.
